# Causal knowledge promotes behavioral self-regulation: An example using climate change dynamics

**DOI:** 10.1371/journal.pone.0184480

**Published:** 2017-09-07

**Authors:** David K. Sewell, Peter J. Rayner, Daniel B. Shank, Sophie Guy, Simon D. Lilburn, Saam Saber, Yoshihisa Kashima

**Affiliations:** 1 Melbourne School of Psychological Sciences, The University of Melbourne, Melbourne, Australia; 2 School of Psychology, The University of Queensland, St. Lucia, Australia; 3 School of Earth Sciences, The University of Melbourne, Melbourne, Australia; 4 Department of Psychological Science, Missouri University of Science and Technology, Rolla, United States of America; 5 School of Health Sciences, University of South Australia, Adelaide, Australia; Universidad de Granada, SPAIN

## Abstract

Adopting successful climate change mitigation policies requires the public to choose how to balance the sometimes competing goals of managing CO_2_ emissions and achieving economic growth. It follows that collective action on climate change depends on members of the public to be knowledgeable of the causes and economic ramifications of climate change. The existing literature, however, shows that people often struggle to correctly reason about the fundamental accumulation dynamics that drive climate change. Previous research has focused on using analogy to improve people’s reasoning about accumulation, which has been met with some success. However, these existing studies have neglected the role economic factors might play in shaping people’s decisions in relation to climate change. Here, we introduce a novel iterated decision task in which people attempt to achieve a specific economic goal by interacting with a causal dynamic system in which human economic activities, CO_2_ emissions, and warming are all causally interrelated. We show that when the causal links between these factors are highlighted, people’s ability to achieve the economic goal of the task is enhanced in a way that approaches optimal responding, and avoids dangerous levels of warming.

## Introduction

An ongoing challenge facing society is how to minimize the negative impacts of global climate change. As noted by Newell, McDonald, Brewer, and Hayes[1], tackling this problem requires an understanding of how people think and reason about (1) environmental systems, and (2) the (adverse) effects that human activities have on these systems. A key insight is that the problem of climate change mitigation can be understood in terms of the more general problem of how people manage and control dynamic causal systems[2]. Osman[3] provided a recent review of the multitude of tasks that have been developed to investigate how people interact with, and manage, dynamic systems, and the different classes of theories that have been developed to explain performance. A common finding is that people often lack insight into the rules, or interactions among variables, that govern how these systems behave. It follows that the *mental models*—the psychological representation of system variables and their effects—people rely on to complete these tasks are lacking in important ways[4]. Generally speaking, people’s mental models of complex systems inaccurately represent and/or relate variables to one another[5], are missing important mechanistic relationships[6], and misrepresent the underlying system dynamics[7]. It follows, then, that people’s ability to control causal dynamic systems tends to be quite poor[3]. A practical challenge lies in identifying ways in which people’s reasoning about causal dynamic systems can be improved, such that better management and control outcomes can be achieved.

The need to improve the accuracy of people’s mental models is especially important for climate change mitigation. Several authors view an accurate mental model of climate change as a necessary step toward adoption of effective mitigation policies[8–9]. However, it is known that laypeople possess highly inaccurate mental models about the causes of climate change[10–13], and that they do not accurately represent the stock-and-flow dynamics that govern the accumulation of atmospheric CO_2_[14]. Because misconceptions about climate change dynamics have been argued to encourage complacency with respect to climate change mitigation[15], a goal of recent research has been to identify ways of overcoming the limitations of people’s mental models to improve their understanding of climate change dynamics. These studies have focused on improving people’s reasoning about accumulation dynamics in tasks with an explicit environmental goal: to stabilize levels of atmospheric CO_2_[14,16–19]. In this article, we build upon this work, by investigating performance in a novel repeated-decision task, where people have an explicit *economic* goal to achieve—rather than an environmental goal—but their progress toward that goal can be hindered by negative effects of global warming within the system. We show that people’s ability to achieve the economic goal is tied to receiving accurate causal knowledge about the system variables. Causal knowledge about the interactions between economic and climate variables within the system appears to bias people toward more cautious means-ends pursuit of the economic goal. The effect of causal knowledge appears to be robust over time and incremental changes in the state of the causal system, and persists for over two hundred consecutive decisions.

We proceed by reviewing previous studies that have examined people’s ability to manage accumulation dynamics in climate change tasks. We discuss some of the methodological limitations of these studies before introducing our novel repeated-decision task. We then consider how a naïve decision-maker might approach the task, based on causal model-based, exemplar-based and hypothesis-testing perspectives on how people manage dynamic causal systems, and consider how causal knowledge might affect performance through the lens of these theoretical frameworks.

### Accumulation dynamics and climate change: Previous studies

It is now well established that people often have difficulty controlling and accurately predicting the behavior of systems that are defined by accumulation dynamics[7,20]. In these systems, the quantity of a stock is affected by inflows and outflows to the system, respectively increasing and decreasing the quantity of stock. As noted repeatedly by Sterman and colleagues, accumulation dynamics of this kind are central to understanding climate change mitigation, as accumulated CO_2_ can be viewed as a stock affected by inflows (i.e., anthropogenic CO_2_ emissions), as well as outflows (i.e., natural carbon sinks, such as forests and oceans). Because warming is driven by the accumulation of atmospheric CO_2_ emissions (i.e., whenever the rate of inflow is greater than the rate of outflow), it follows that climate change mitigation hinges, minimally, on stabilization of the stock of atmospheric CO_2_.

Sterman and Booth Sweeney[14] introduced a *climate stabilization task* intended to assess how people reason about accumulation dynamics applicable to climate change mitigation. People were shown graphs illustrating the stabilization of atmospheric CO_2_ (the stock) at some time in the future. People were also shown a graph of the recent history of CO_2_ emissions (the inflow) along with the current rate at which CO_2_ was removed from the atmosphere (the outflow). The task was to sketch trajectories of emissions and passive CO_2_ removal—the latter could effectively be assumed to be constant—that would achieve stabilization of the stock illustrated in the first graph. The results of this task showed that people frequently failed to identify that stabilization of the stock could only be achieved when the rate of inflow (CO_2_ emissions) matched the rate of outflow (passive removal of atmospheric CO_2_). Instead, people’s projected emissions rates consistently exceeded the rate at which CO_2_ was removed, highlighting a failure to understand the fundamental accumulation dynamics that drive global warming.

Subsequent studies have sought to improve people’s performance on the climate stabilization task by providing analogies to assist people’s reasoning. For example, Guy, Kashima, Walker, and O’Neill[17] likened accumulation of atmospheric CO_2_ to water filling a bathtub, and found that presentation of the bathtub analogy reduced the extent to which people overestimated emissions rates needed to stabilize CO_2_ levels. The effectiveness of analogy is not unconditional, however, and depends on a number of other factors, such as level of education[17], the presence or absence of graphical displays of inflows and outflows[17,19], as well as the specific content of the analogy presented[18–19].

### Extending previous research

A limitation of previous climate stabilization studies, such as those by Guy et al. [17], Newell et al. [19], and Sterman and Booth Sweeney[14] is that people were not provided with an opportunity to observe the accumulation dynamics of the system by interacting with it. Moxnes and Saysel[18] investigated a version of the climate stabilization task where people could observe the effects of emissions on CO_2_ accumulation over successive 10-year intervals. They found that people who were able to observe the system dynamics in this way performed better than people who did not have an opportunity to observe the effects of emissions on accumulation. Similarly, Dutt and Gonzalez[21] investigated the effect of observing the system dynamics, via interacting with the system, on subsequent performance on the climate stabilization task. They introduced an iterated version of Sterman and Booth Sweeney’s[14] CO_2_ accumulation scenario, where people were required to enter values that set rates of CO_2_ emissions and removal of CO_2_ on a trial-by-trial basis to track the trajectory of the accumulated stock of atmospheric CO_2_ through time. People who had the opportunity to observe the accumulation dynamics by interacting with the system performed better on the climate stabilization task compared to people who did not get a chance to interact with the system beforehand.

A more detailed examination of the way feedback about accumulation dynamics affects people’s ability to manage a (simulated) climate system was reported by Dutt and Gonzalez[16]. They presented people with an iterated version of the climate stabilization task, requiring participants to keep CO_2_ concentration within a particular range of values on a trial-by-trial basis. When CO_2_ levels exceeded the acceptable range for that trial, people incurred an economic penalty—which affected whether they received a monetary bonus at the end of the experiment—and thus had an opportunity to receive fine-grained feedback about their performance in the task. This study found that people’s ability to stabilize the climate system over the long-term was improved when feedback was more frequent and when system variables respond faster to changes in emissions levels. The latter echoes earlier findings that people often struggle to stabilize dynamic systems that incorporate time delays and feedback loops[22–23].

A common feature of previous research has been the use of tasks that include an explicit climate stabilization goal. In all of the key studies reviewed thus far, people were specifically tasked with identifying a level of CO_2_ emissions that will stabilize the stock of atmospheric CO_2_. This focus potentially ignores the role that economic factors play in shaping the stances that individuals (and nations) are likely to adopt in relation to climate change mitigation. At the level of individuals, economic concerns about wages and job availability heavily influence people’s views about climate change intervention[24]. At the national level, economic down turns are often associated with a decline in public concerns about climate change[25–26]. Similarly, national and international decisions about climate change mitigation will be strongly influenced by the estimated costs of climate change mitigation[27]. It follows that understanding how people make decisions about managing the earth’s climate will be informed by, and perhaps principally decided by, economic considerations.

### Current study

In this article, we examine how people interact with a (simulated) climate system when their goal is economic, rather than environmental, in nature. We further examine the effect causal knowledge about the system variables has on people’s ability to achieve the economic goal. To this end, we introduce a novel iterated decision task, where economic decisions are directly linked to environmental outcomes, which, in turn, affect people’s ability to achieve an economic goal. Like previous studies that have used iterated decision tasks[16,21], the responses made by people in our task drive changes in the state of the climate system. That is, people’s chosen actions produce emissions that affect the accumulated stock of atmospheric CO_2_, and the level of global warming. Unlike previous research, the actions that people in our study take are *economic actions*, rather than environmental actions. Instead of setting levels of emissions directly—as in previous climate stabilization tasks—people’s responses drive changes to an economy they are managing. In our task, people’s economic actions are not tied to an explicit environmental goal. Participants are not instructed to stabilize CO_2_ levels, rather, their goal is to grow the economy such that it is at least doubled by the end of the task. However, the climate and economic systems in our task are linked such that increases in warming make it progressively more difficult to achieve economic growth. By providing people with an explicit economic goal, our task incorporates, in a novel way, a tension between achieving economic growth on the one hand, and managing CO_2_ emissions on the other.

### Task overview: Managing a dynamic human-climate system

**Fig 1:**
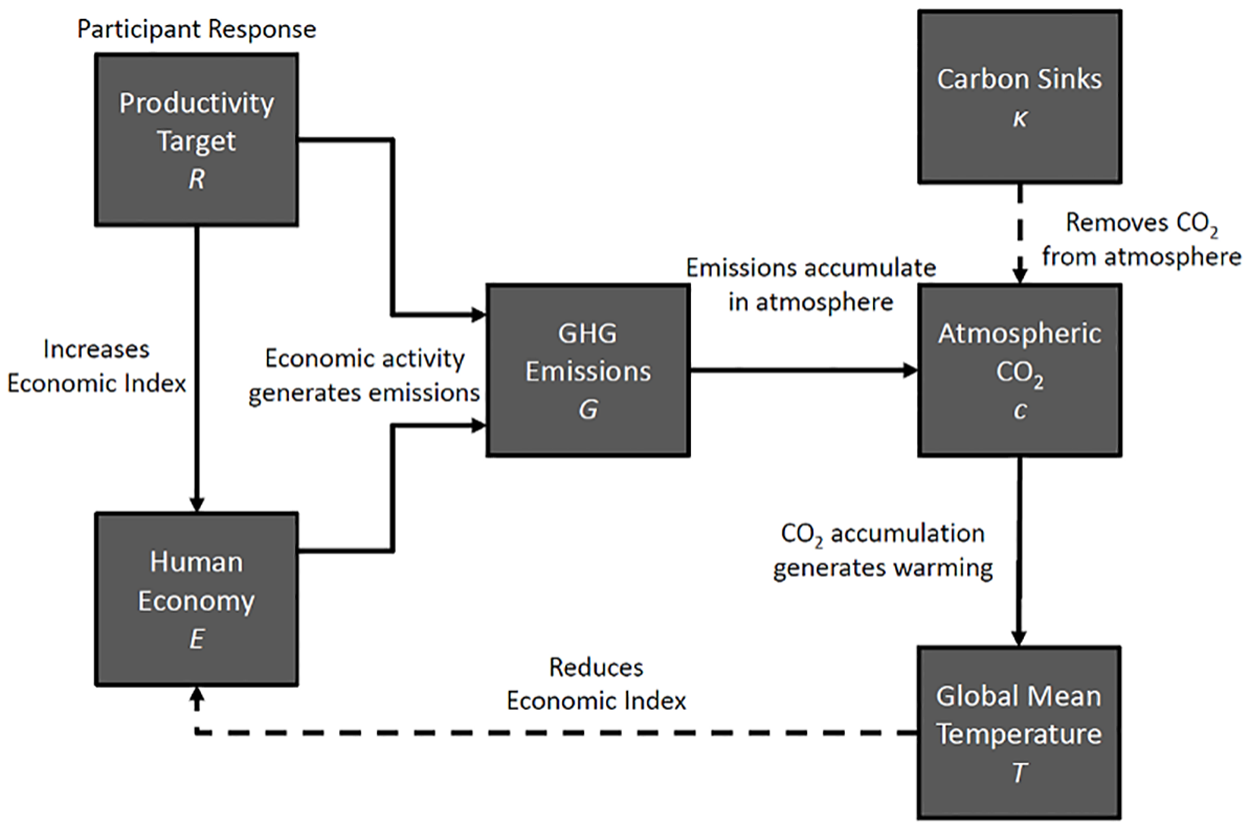
Schematic of the interactive human-climate model. Participants set an economic productivity target, *R*, which drives economic growth, *E*, but also generates greenhouse gas emissions, *G*. Emissions accumulate in the atmosphere, subject to passive removal by natural carbon sinks at rate *κ*. CO_2_ accumulation, *c*, generates warming, increasing the global mean temperature, *T*. Temperature has a negative effect on economic growth. Capacity for economic growth is reduced under increased warming.

In our task, people adopt the role of a policy director setting annual economic targets, with an explicit goal to double the size of the economy by the end of the experiment. On each trial in the experiment, people are shown numerical values reporting the current size of the economy, concentration of atmospheric CO_2_, and global mean temperature. The participant then sets an economic growth target, which updates the size of the economy, CO_2_ concentration, and temperature. The relationship between variables in the human-climate system used in our task is summarized schematically in Fig 1.

**Fig 2:**
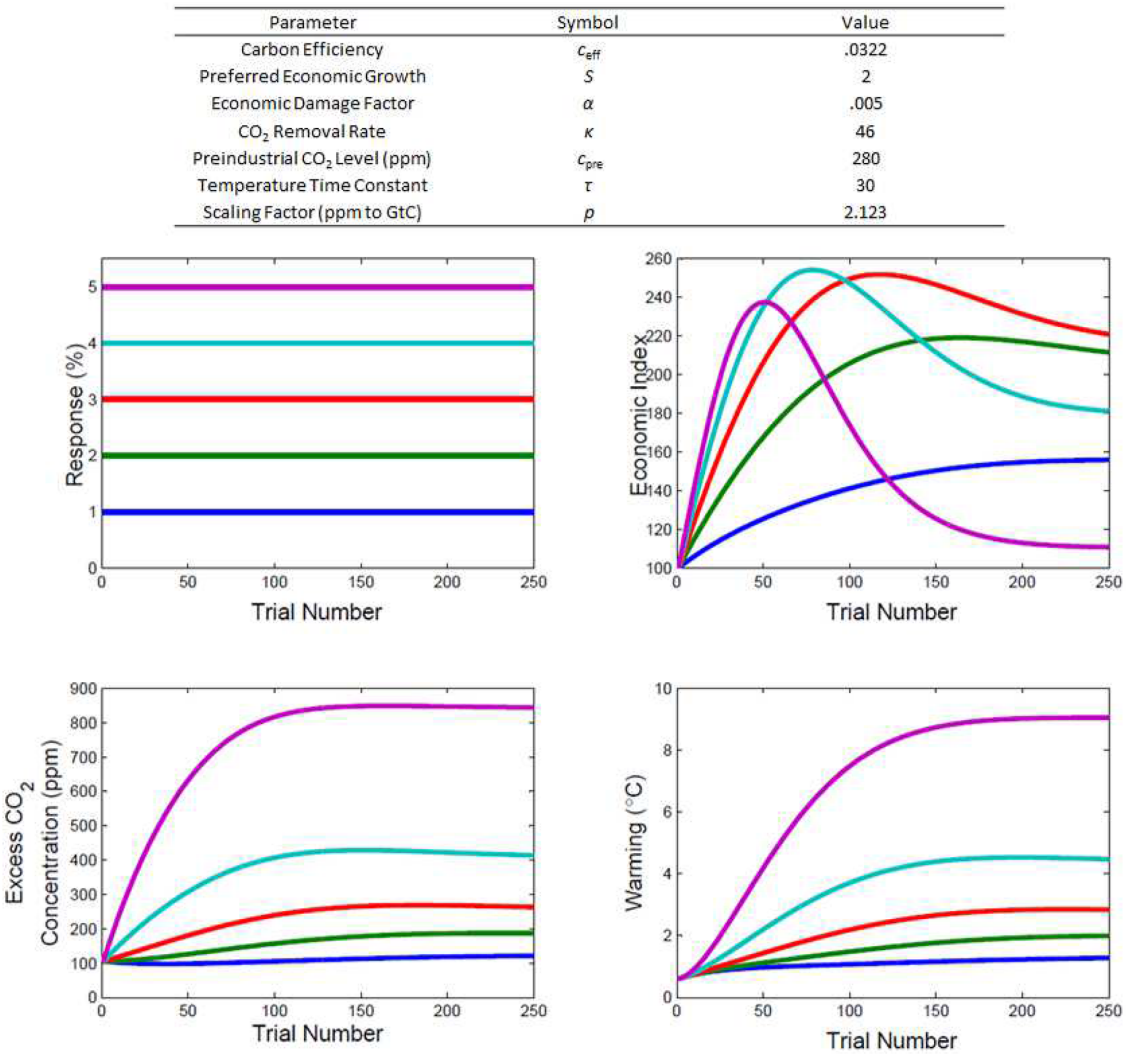
Illustration of the human-climate model dynamics for five different levels of responding. Economic productivity targets of 1, 2, 3, 4, and 5 percent are shown by the blue, green, red, cyan, and purple solid lines, respectively. Parameters governing the behavior of the model are shown above the panels.

Formally, the changes in system variables are governed according to the following system of equations, based on the MAGICC intermediate complexity Earth system model[28], which describes the relationship between emissions, atmospheric CO_2_ concentration, and temperature. On each trial of the experiment, indexed by *i*, the participant generates a response, *R*_*i*_, which sets an economic growth target. The resulting economic activity generates a quantity of greenhouse gas emissions, *G*_*i*_, according to

$$G_{i}=\left[E_{i}+\text{exp}\left(R_{i}+s\right)\right]c_{\text{eff}},$$ (1)

where *E*_*i*_ is the size of the economic index on trial *i*, *s* is a scaling parameter indexing preferred growth, and *c*_eff_ is a carbon efficiency constant. Fig 2 lists values for constants used in the model—these fixed parameter values were informed by recent calibration studies of the MAGICC model against other climate models[28] and data[29]. The change in the economic index in light of the response, *R*_*i*_, is given by

$$\Delta E_{i}=R_{i}-\alpha T_{i}E_{i},$$ (2)

where *T*_*i*_ is the level of warming on trial *i*, and *α* is an economic damage constant that determines the negative effect of warming on the economic index. A minimum value of 0 was enforced when implementing trial-by-trial changes to the economic index.

Changes in emissions drive changes in atmospheric CO_2_ concentration according to

$$\Delta c_{i}=\frac{G_{i}}{p}-\frac{c_{i}}{\kappa },$$ (3)

where *c*_*i*_ is the level of atmospheric CO_2_ on trial *i*, *p* scales parts per million to gigatons of carbon, and *κ* is the rate at which CO_2_ is removed from the atmosphere by passive carbon sinks. Eq 3 therefore summarizes the stock-and-flow relationship between atmospheric GHGs, anthropogenic emissions, and carbon sinks in the environment.

Changes in temperature provoked by changes in CO_2_ concentration are given by

$$\Delta T_{i}=\frac{\sigma c_{i}}{c_{\text{pre}}}-\frac{T_{i}}{\tau },$$ (4)

where *σ* indexes the climate sensitivity, the effect doubling CO_2_ concentration over pre-industrial levels, *c*_pre_, would have on global mean temperature. The parameter *τ* reflects natural time delays associated with changes in global mean temperature.

The behavior of the human-climate model is summarized in Fig 2. The panels illustrate the dynamics of the economic index, CO_2_ concentration, and warming under five different levels of responding—for simplicity, constant economic targets are used in the figure.

To get intuition about the behavior of the system, it is useful to consider how changes in responding affect levels of CO_2_, warming, and the economic index. Fig 2 shows that there are strict order relations between responding and both CO_2_ and warming—higher levels of economic activity result in increased emissions, and, in turn, increased warming. It is also clear from the figure that these order relations do not hold for the economic index. Owing to the harmful effect of warming on economic performance (Eq 2), the relationship between responding and the economic index is nonmonotonic. Although setting high growth targets early in the task generates substantial economic growth, the high level of emissions associated with this response pattern makes it such that, toward the end of the task, high levels of warming prevent economic growth, leading to subsequent declines in the economic index. By contrast, lower productivity targets result in smaller, but sustained, economic growth over the duration of the task.

Several other properties of the human-climate system are especially noteworthy. First, the system is defined by time delays and multiple non-linear interactions among variables that are not visible to the participant. These factors make it virtually impossible to predict the quantitative behavior of the system through observation alone. Although people can uncover simple causal structure—usually involving linear, but probabilistic, relations among variables with no time delays—quite well through analyzing the effects their behavioral interventions have on the system[30–32], people’s ability to predict and control systems with more complex dynamics, like our human-climate system, can be quite poor[3,22–23]. Because our principal experimental manipulation involves providing some, but not all, participants with causal knowledge about the relationship between economic activities, CO_2_, and warming, having a low expected baseline level of performance is advantageous. If causal knowledge from the outset of the task confers a performance benefit, there should be ample room to observe its effect in the data.

The second noteworthy aspect of our task has to do with the presence of time delays within the system[16]. In our task, people are able to adjust their responding across successive trials to adapt to changes in the system state during the task. However, time delays in the system—particularly with regards to temperature (Eq 4)—mean that, by the time the harmful effects of warming on economic growth become apparent, even quite radical changes in responding may not be enough to counteract the problem. Once warming increases past a certain point, no level of responding will result in economic growth (Eq 2). A key question is whether people are able to avoid this “point of no return” without causal knowledge of the relationship between the economic and climate variables in the system.

### Ways of approaching the task: Effects of causal knowledge

How might individuals respond in our task to achieve the economic goal of doubling the size of the economy by the end of the experiment? How might people’s response strategies be affected by causal knowledge of the system variables? Because people in our task have an explicit economic goal to achieve (i.e., doubling the size of the economy) and a clear means of achieving that goal (i.e., responding by setting large economic growth targets), we assume that people will tend to adopt a means-ends approach to the task. That is, we expect people to set economic growth targets that will promote rapid growth of the economic index, given the absence of relevant causal knowledge. Because of the time delays involved in warming driven by accumulated CO_2_ (Eqs 3 and 4), this pattern of responding will promote positive interim outcomes—rapid progress toward achieving the economic goal of the task. Accordingly, it would be predicted on the basis of instance theories[33], hypothesis-testing perspectives[34], as well as causal model-based theories[35], that people would favor responding in this way for the duration of the task. Once warming increases to the point where economic growth is harmed—or even prevented—the predictions from these theories become less clear. Under instance theory, people will not have a bank of similar trial instances to retrieve once economic growth targets fail to achieve growth on a given trial, and so it is possible that people might persist in setting high growth targets. Under a hypothesis-testing framework, people may similarly be expected to persist in setting high growth targets, as doing otherwise would result in moving people away from achieving the economic goal (i.e., setting *negative* growth targets would reduce the economic index). However, because high levels of warming would prevent even high growth targets from increasing the economic index (Eq 2), people might attempt to change their response pattern by inferring from feedback, that their current strategy is ineffective. Under a causal model-based approach, we would expect people to have developed a mental model of the task that incorporates a strong positive relationship between growth targets and approach toward the economic goal. Accordingly, people would be expected to persist in setting high growth targets even after high levels of warming begin to limit increases to the economic index (Eq 2).

When people have access to causal knowledge about the relationship between the climate and economic variables in the system, we expect people to still apply a straightforward means-ends approach to the task. However, we predict that people would adopt a more conservative response profile, where relatively lower economic growth targets are set at the outset and persist at relatively low levels for the duration of the task. The expected effect of causal knowledge is perhaps most readily understood in terms of hypothesis-testing approaches[34] and causal model-based perspectives[35], as people would be aware that allowing warming to increase would have a strong negative effect on their ability to achieve the economic goal. It follows that under both of these frameworks, people would be predicted to set more conservative economic growth targets in an effort to avoid the harmful effects of warming on their ability to grow the economic index. In sum, we expect that people will not manage the task well unless they are provided with causal knowledge about the system variables at the outset of the task.

### Overview of experiments

Our principal experimental manipulation involves a knowledge-based intervention where the causal relationship among economic activities, CO_2_, and warming variables, is (Informed condition), or is not (Uninformed condition) explicitly revealed to people at the outset of the task (see S1 Text for details). Contrasting performance across the two conditions reveals whether causal knowledge affects people’s ability to achieve the economic goal in light of the negative effects of warming. Given that people are known to perform poorly in tasks involving non-linear systems with time delays[22–23], we expect causal knowledge to bias people’s interactions with the system such that they favor more conservative responding. This will, in turn, improve their ability to achieve the economic goal of the task and avoid harmful effects of warming.

We were also concerned that the labels for the system variables—specifically, CO_2_, and warming—might introduce a demand effect, especially among our university sample in Experiment 1, that could artificially bias people toward more conservative responding. To determine whether any benefits of causal knowledge were restricted to variables involved in climate change, we ran a separate version of the task using a different cover story involving fictitious bacterial populations. Importantly, the system dynamics—based on known accumulation dynamics involved in the carbon cycle—were identical across the two versions of the task (i.e., governed by Eqs 1–4). However, the labels for the system variables were different. Given that knowledge effects are frequently found using a variety of cover stories in the causal learning literature[31,35–37], we expected similar results across the different cover story conditions.

Across two experiments, using a university student sample and a sample of the general public, we show that causal knowledge improves people’s ability to achieve the economic goal of our task whilst avoiding (economically) harmful levels of warming. We show that the beneficial effect of causal knowledge is not restricted to the climate change domain: When the same system dynamics were presented in the context of managing a population of bacteria, the same pattern of effects emerged, ruling out experimental demand characteristics and ideological views on climate change as explanations for our data. Finally, we show that, when provided with causal knowledge, responding approaches what could be considered an optimal profile for the task.

## Experiment 1: Australian university sample

### Method

#### Ethics statement

Both experiments were approved by and conducted in compliance with the University of Melbourne Human Research Ethics Committee. After reading an information sheet describing the task, participants in Experiment 1 signed and returned a consent form to the experiment. In Experiment 2, which was conducted online, participants read through an information screen prior to commencing the task, and indicated their consent by agreeing to proceed from the information screen to the task itself via mouse click. All consent procedures were approved by the University of Melbourne Human Research Ethics Committee.

#### Design and participants

The experiment was a 2 (Information Condition: Informed vs. Uninformed) × 2 (Cover Story: Climate vs. Bacteria) between-subjects design. One hundred first-year psychology students from the University of Melbourne were recruited in exchange for course credit.

#### Materials and procedure

Participants were randomly allocated to one of the four experimental conditions. All participants were provided with information describing the key features of the task display and instructions that their goal was to maximize the Economic Index (or Bacterial Population), and at least double it by the end of the experiment. Participants in the informed conditions were provided with additional information describing the causal relationships between human economic activity and the three climate variables. The causal knowledge manipulation included the following text summarizing the relationships: “Economic productivity affects CO_2_ concentration, which in turn affects Temperature. Temperature increases make it increasingly difficult to achieve economic growth. Due to time lags in the climate system, the effects of CO_2_ on economic growth will only be felt after a considerable delay, after which they will be difficult to reverse. Hence, it is advisable to keep CO_2_ concentration from escalating too high.” (see S1 Text for complete instructions). The experiment was controlled by a Matlab program designed using the Psychophysics Toolbox. Participants completed the experiment individually on PC computers. The task took approximately 30–40 minutes to complete.

The experiment began once participants had read the summary information about the task. On each trial, participants were presented with a display containing current information about the three task parameters, Economic Index, CO_2_ concentration, and global mean temperature. They then set an economic target by clicking in the response box, which allowed participants to aim to grow or contract the economy by up to five percent in either direction. Once a target had been selected, participants confirmed their response before proceeding. Upon confirmation, the human-climate system variables were updated to take on new values. Participants were presented with the new values along with the relative changes before clicking a button to proceed to the next trial.

### Results

**Fig 3:**
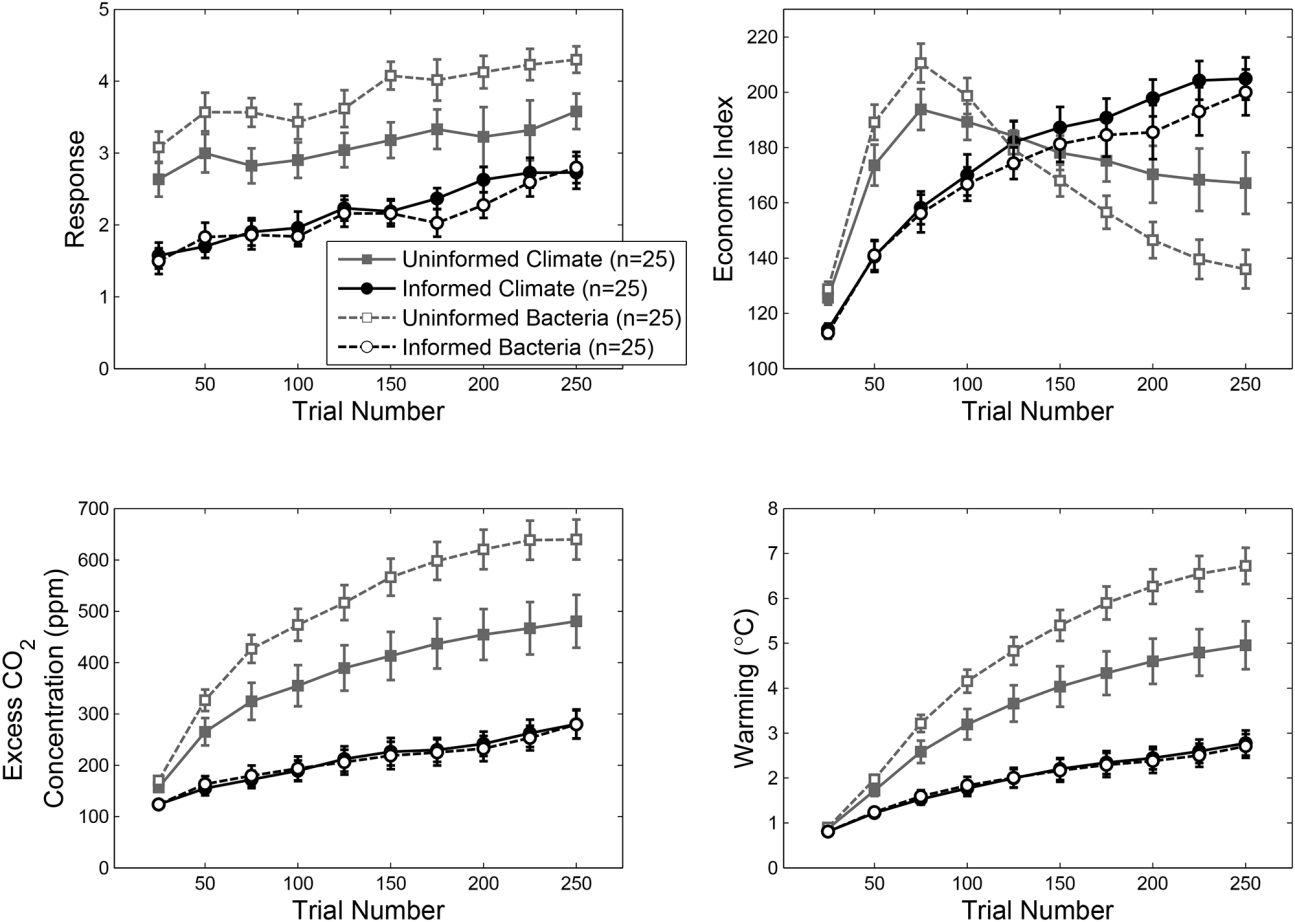
Data from Experiment 1. Panels show group averaged data for participant responses (a), economic index (b), excess CO_2_ concentration (c), and warming (d). Error bars are one standard error of the mean.

The results of Experiment 1 are summarized in Fig 3. For ease of exposition, we describe results and effects using the variables labels from the climate change cover story condition. The four panels of the figure plot trajectories of people’s responses (i.e., economic targets) along with trajectories of the size of the economy, excess CO_2_ concentration, and warming (which are determined by the underlying system dynamics). Data were binned into 10 25-trial epochs, and were averaged across participants within each condition. Data for each variable (response, economic index, CO_2_, and temperature) were analyzed with a 2 (Information Condition: Informed vs. Uninformed) × 2 (Cover Story: Climate vs. Bacteria) × 10 (Trial Epoch) between-within ANOVA. Because of the large number of variables analyzed, we restrict our discussion to the most relevant effects (see S1 Text for additional analyses).

Two clear patterns are evident in the economic targets selected by participants over the course of the experiment (Fig 3A). First, participants in the Informed condition set more conservative economic targets than participants in the Uninformed condition, *F* (1, 96) = 50.41, *MS*_*e*_ = .001, *p* < .001, *η*_*p*_^*2*^ = .34. Second, economic targets increased over the course of the experiment, *F* (9, 864) = 24.42, *MS*_*e*_ = 1e-5, *p* < .001, *η*_*p*_^*2*^ = .20 (Fig 3A), indicating that (a) participants were attempting to grow the economy to meet the explicit goal of doubling the size of the economy by the end of the experiment, and (b) participants responded to the increased difficulty in achieving economic growth—because of the negative effect of warming on economic growth (Eq 2)—by progressively setting higher growth targets. A trend analysis showed that the changes in economic targets increased linearly across trial epochs, *F* (1, 98) = 83.87, *MS*_*e*_ < .001, *p* < .001, *η*_*p*_^*2*^ = .46. No higher order trends were significant. Turning to the follow-on effects of participant responses on the economic index and the two environmental variables, there are distinct patterns characteristic of the Informed and Uninformed conditions. For the Uninformed participants, the economic index initially increased rapidly, but then declined toward the end of the experiment. For the Informed participants, the economic index steadily grew throughout the experiment. The difference between the economic trajectories was supported statistically by an interaction between Information Condition and Trial Epoch, *F* (9, 864) = 38.53, *MS*_*e*_ = 772.34, *p* < .001, *η*_*p*_^*2*^ = .29. Note also that Informed participants were more successful at meeting the primary goal of the task, doubling the economic index by the final Trial Epoch, in both the Climate, *t* (48) = 2.80, *p* = .007, *r*^*2*^ = .14, and Bacteria, *t* (48) = 5.89, *p* < .001, *r*^*2*^ = .42, Cover Story conditions.

Similar patterns of differences between the Informed and Uninformed conditions emerged for the CO_2_ and temperature variables—irrespective of Cover Story—as shown in Fig 3C and 3D. Uninformed participants achieved poorer environmental outcomes than Informed participants, reflected in main effects of Information Condition for both CO_2_, *F* (1, 96) = 66.80, *MS*_*e*_ = 193860.94, *p* < .001, *η*_*p*_^*2*^ = .41, and temperature, *F* (1, 96) = 66.14, *MS*_*e*_ = 16.23, *p* < .001, *η*_*p*_^*2*^ = .41.

### Discussion

The results of Experiment 1 are readily summarized. Regardless of cover story, providing people with knowledge of the causal relations among system variables improved people’s ability to perform the task. The benefit of receiving this information up front is particularly interesting, as learning by observing the effects of responses often results in successful performance[30,32]. We believe that the complexity of our task, specifically, the incorporation of non-linear dynamics and time delays contributed to the causal knowledge effect we observe here, as learning the precise behavior of the system through the effects of individual responses alone would be extremely difficult, if not impossible. That the benefits of causal knowledge were sustained across both cover story contexts implies that artificial demand characteristics or specific views about climate change were not key drivers of performance in our task.

## Experiment 2: United states general population sample

To generalize the Experiment 1 results to a broader population, we conducted an online replication study with a US sample recruited from the general population.

### Method

#### Design and participants

The experimental design was the same as in Experiment 1. Participants for Experiment 2 were recruited online via Amazon Mechanical Turk, which has been shown to replicate a multitude of benchmark results involving complex, multi-trial experimental designs like ours[38]. Participant samples recruited via Mechanical Turk are known to be demographically more diverse than university samples[39]. In total, 112 participants completed the experiment and were paid $3 in exchange for their participation. The remuneration rate was commensurate with other psychological studies that have used Mechanical Turk for participant recruitment purposes.

#### Materials and procedure

The general procedure was identical to that of Experiment 1, except that the experiment was controlled using Python/Django routines for online presentation purposes.

**Fig 4:**
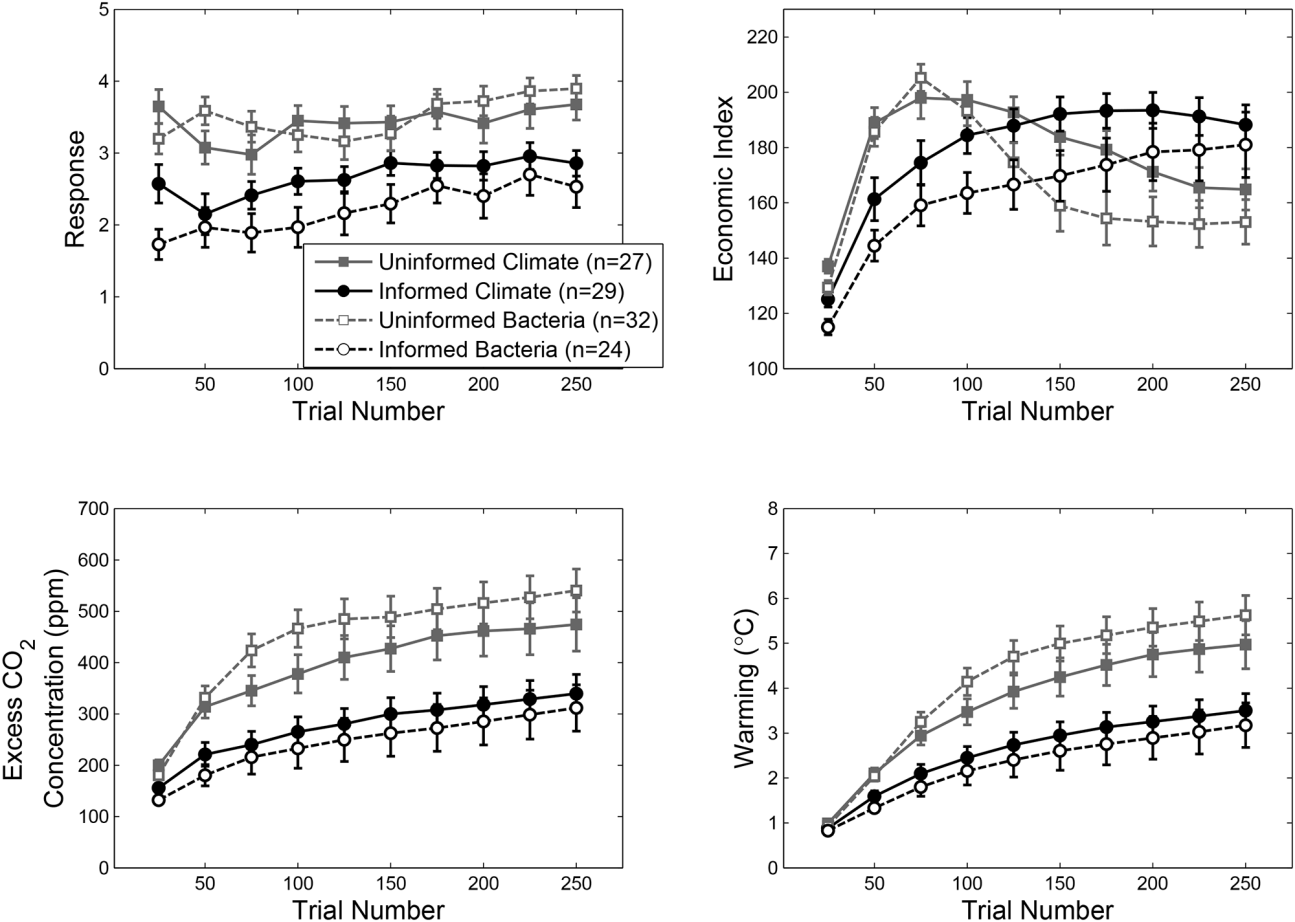
Data from Experiment 2. Panels show group averaged data for participant responses (a), economic index (b), excess CO_2_ concentration (c), and warming (d). Error bars are one standard error of the mean.

The data from Experiment 2, presented in Fig 4, mirror those from Experiment 1. As in Experiment 1, economic targets selected by participants increased over the course of the task, *F* (9, 972) = 10.13, *MS*_*e*_ = 1e-6, *p* < .001, *η*_*p*_^*2*^ = .09 (Fig 4A). A trend analysis confirmed that the increase in economic targets was linear across trial epochs, *F* (1, 110) = 28.46, *MS*_*e*_ < .001, *p* < .001, *η*_*p*_^*2*^ = .26. There were also significant, but less readily interpretable, cubic, *F* (1, 110) = 6.53, *MS*_*e*_ = 5e-5, *p* = .012, *η*_*p*_^*2*^ = .06, and 8^th^-order polynomial trends, *F* (1, 110) = 5.08, *MS*_*e*_ = 3e-5, *p* = .026, *η*_*p*_^*2*^ = .04. No other trends were significant. Critically, there was an effect of Information Condition reflecting lower, more conservative, economic targets being set by people in the Uninformed condition relative to the Informed condition, *F* (1, 108) = 28.90, *MS*_*e*_ = .001, *p* < .001, *η*_*p*_^*2*^ = .21.

The economic consequences of differential responding in the Informed and Uninformed conditions are illustrated in Fig 4B. As in Experiment 1, Uninformed participants produced a “boom-bust” economic trajectory, characterized by a sharp initial rise in the economic index followed by decline over the latter portion of the task. By contrast, Informed participants increased the economic index throughout the task, resulting in a significant interaction between Information Condition and Trial Epoch, *F* (9, 972) = 18.60, *MS*_*e*_ = 882.46, *p* < .001, *η*_*p*_^*2*^ = .15. Like Experiment 1, participants in the Informed conditions were more successful at achieving the economic goals of the task by the final Trial Epoch in both the Climate, *t* (54) = 2.27, *p* < .03, *r*^*2*^ = .09, and Bacteria, *t* (54) = 2.02, *p* < .05, *r*^*2*^ = .07, Cover Story conditions.

Experiment 2 produced CO_2_ and temperature trajectories that strongly resembled those found in Experiment 1 (see Fig 4C and 4D). Uninformed participants produced poorer environmental outcomes than Informed participants. Mirroring Experiment 1, there were main effects of Information Condition for both CO_2_, *F* (1, 108) = 23.65, *MS*_*e*_ = 299014.48, *p* < .001, *η*_*p*_^*2*^ = .18, and temperature, *F* (1, 108) = 24.33, *MS*_*e*_ = 24.84, *p* < .001, *η*_*p*_^*2*^ = .18.

### Discussion

The results of Experiment 2 replicate those of Experiment 1 with a general population sample. We again demonstrate that the beneficial effects of causal knowledge are robust across cover stories, implying that experimental demand effects did not have a strong influence over performance in our task.

## Optimal response analysis

Our analysis of the behavioral data showed that providing people with information about the causal relationships between human economic activities, CO_2_ accumulation, and warming, improved their ability to manage the human-climate system. Given the explicit economic goals of the task though, a pertinent question is how well people *could* perform in our task, given the instructions. To this end, we conducted an optimal response analysis. The aim of the analysis was to identify the response profile that maximized the average of (1) the mean economic index over the course of the task, and (2) the economic index at a time point beyond the life of the task (i.e., at trial 300). These criteria map onto the instructions to (1) maximize the overall economic index, and (2) aim to at least double the size of the economy by the end of the task. For the latter criterion, evaluating the size of the economy at trial 300—rather than trial 250—was chosen to reduce the influence of end-of-task artifacts associated with optimization (e.g., excess warming will not penalize economic growth immediately due to time delays in the human-climate system, which means that the optimal way to respond on the last 20–30 trials is to set the highest possible economic growth targets; see S1 Text for complete details).

For the optimal response analysis, we adopted a parameter estimation approach. For our initial analyses, we divided the task into 25 equally-sized response intervals (i.e., for 300 trials, each response interval comprised 12 trials). Using a standard simplex algorithm, we estimated, for each interval, an economic target from the range of responses available to participants (i.e., the interval of -5 to +5), which maximized the joint optimization criteria. Our initial analyses revealed that much of the estimated optimal response functions had a clear exponential form. We also found that across different optimization runs, estimates for the early response intervals were quite volatile. This makes sense, as the effects of the earliest responses can, to some extent, be offset by later ones, given the optimization criteria we used. To improve stability and better avoid local minima—and greatly reduce the time required for optimization—we considered an exponential response function,

$$R_{i}=\alpha \text{exp}\left(ci\right)+\Delta .$$ (5)

**Fig 5:**
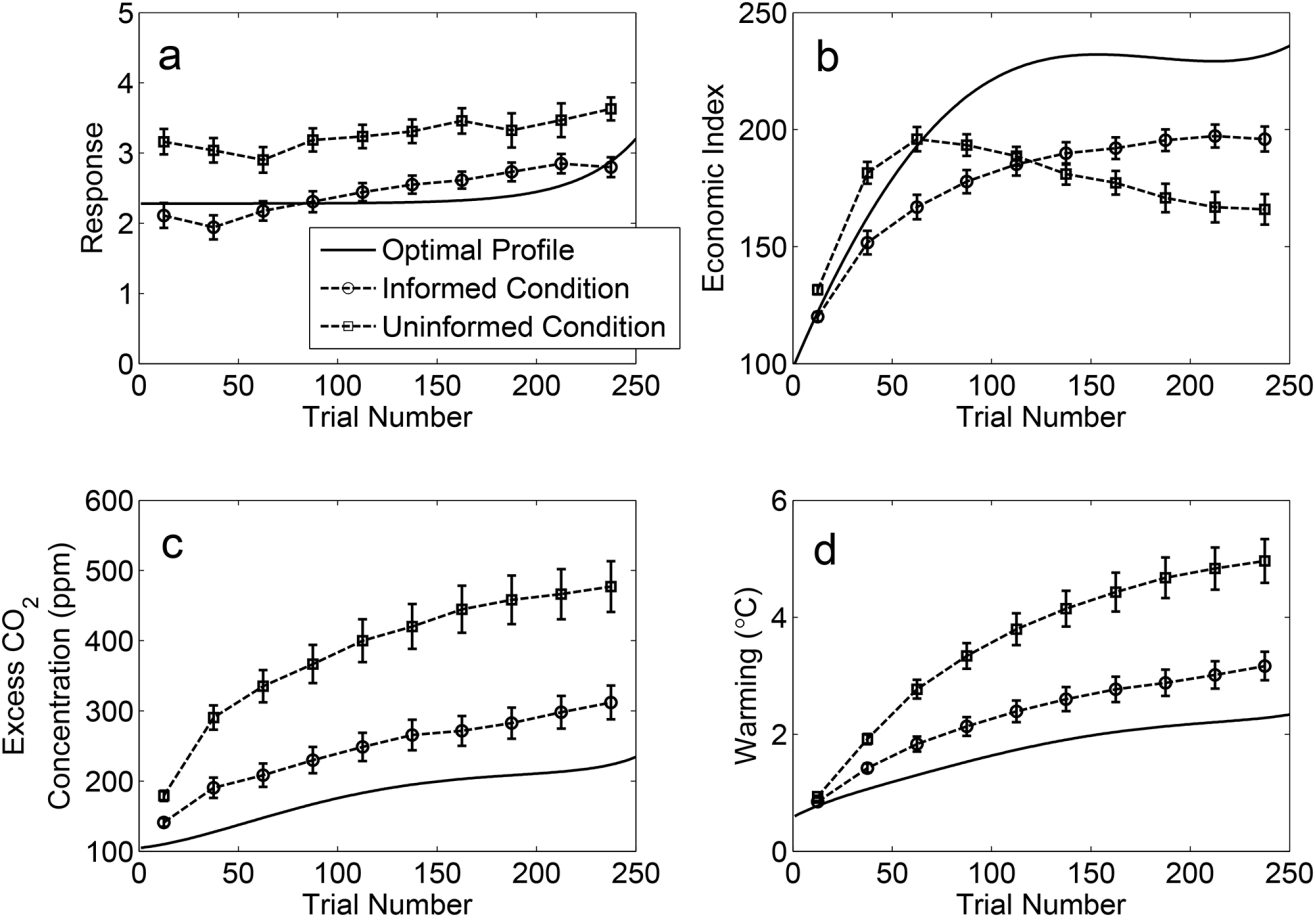
System dynamics associated with the optimal response profile (solid curves) overlaid with data from informed and uninformed conditions (dashed lines). Data are collapsed across cover story conditions and across Experiments 1 and 2. The panels show trajectories for responses (a), economic index (b), excess CO_2_ concentration (c), and warming (d). Error bars are one standard error of the mean.

In Eq 5, *R*_*i*_ denotes the response, or economic target set, on trial *i*, *α* is scaling parameter, *c* determines the steepness of the exponential function, and *Δ* is an offset term. The optimal response profile, along with the resulting economic and climate dynamics, is shown in Fig 5 alongside response trajectories and system dynamics for the informed and uninformed conditions—averaged across both experiments and cover story conditions.

The optimal response profile is characterized by setting moderate economic growth targets for much of the task—approximately +2.25—before progressively increasing responding toward the end of the experiment. Although this ramping up of economic growth targets partially reflects an “end-of-task” artifact—due to time lags in the human-climate system, the warming that would result from the increase in economic activity toward the end of the task does not have sufficient time to provoke a reduction in the economic index (see Eqs 2 and 4)—participants in our experiments also tended to increase economic growth targets in the latter part of the task. The most striking aspect of the optimal response profile is in how similar it is to performance of the informed participants over the course of the task. One implication is that causal knowledge may lead people to behave in a way that is not only sustainable, but more closely approximates optimal management of the human-climate system. This is consistent with the idea that causal knowledge results in improved, but imperfect, performance in a variety of causal inference tasks[40].

## General discussion

A fairly common view among researchers is that adoption of effective climate change mitigation policies depends on people having an accurate mental model of the processes that lead to global warming[8–9]. However, people have repeatedly been shown to struggle to understand the fundamental accumulation dynamics that are involved[7,14]. We investigated the effect of providing people with causal knowledge about the variables involved in CO_2_ accumulation and warming in the context of a novel iterated decision task where pursuit of an economic goal drove changes in the climate system. We showed that providing people with up-front information about relevant causal relationships in the human-climate system resulted in improved task performance, suggesting that people successfully used this causal knowledge to guide their responding in the task. The basic pattern of results was replicated in a parallel set of experiments that framed the decision task in terms of managing a fictitious bacterial population, suggesting that the benefits of receiving causal knowledge were not restricted to variables related to climate change. We further showed that receiving causal knowledge about the underlying system resulted in decision-making performance that approached an optimal response profile.

### Limitations

Although our study extended existing work on the effect of knowledge in reasoning about climate change[16,21], it is not without its limitations. We discuss several limitations and potential concerns about (1) distinguishing effects of causal knowledge from response bias, (2) the way economic activities were represented in the human-climate system, and (3) whether our results will scale up from individual decision-makers to collectives.

As noted by reviewers, it is possible that our manipulation of causal knowledge simply induced a favorable response bias in participants in the Informed condition. We acknowledge that, while we cannot completely rule this out as a possible explanation, the fact that people in the Informed condition produced response profiles that adapted to the changing demands of the task—increasing economic productivity targets to potentially compensate for the negative effects of warming on economic growth toward the end of the task (see Figs 3 and 4)—speaks against a simple response bias account. Nevertheless it would be worth investigating whether instruction to simply produce low economic growth targets early on in the task, without providing any other information about how the variables interact, would produce a different response profile in the Informed condition than the causal knowledge intervention we used in the current experiments. One possibility is that promoting a response bias (without causal knowledge) would result in no difference in performance compared to the Informed condition. We suspect, however, that without causal knowledge, people may be reluctant to continue making conservative economic growth targets when the system dynamics—at least in the early stage in the task—provide no compelling reason to do so. Although people will achieve economic growth when responding conservatively, exploration of the effects of responding with higher economic growth targets would reveal that more rapid economic growth could be achieved. Ultimately, this is an empirical question that awaits future research.

Another potential limitation of our study was in the way economic activities were implemented in the human-climate system. Rather than having economic growth being characterized in terms of the aggregate of a number of different kinds of economic activities (e.g., different types of industry with potentially different carbon intensities), we relied on only a single, monolithic, economic sector. We readily acknowledge that this limits the realism of our human-climate system, but note that increasing the complexity of what is already a complex system is likely to result in poor performance, which can create substantial difficulties inferring people’s decision strategies from data[41–42]. Put another way, the (over-)simplified economic aspect of our human-climate system is a feature, rather than a bug. It is nevertheless an interesting question to investigate what effect, if any, providing people with different economic response alternatives will have on people’s ability to manage the human-climate system.

Finally, we note that the current study investigated individual decision-making behavior, whereas the problem of climate change mitigation is unequivocally a problem of *collective* decision-making. We conjecture that providing the public with better information about the underlying causes of climate change—in addition to the strong scientific consensus on the role of CO_2_ emissions—will produce decision-making benefits at the group level. As the proportion of well-informed decision-makers increases, it is reasonable to suspect that the quality of decisions made by aggregations of such agents will also be improved.

At the same time, results that identify highly polarized opinions along political lines implies that provision of relevant information may not produce the desired effects on group decision-making unless the information is presented in a way that resonates with diverse socio-economic values[43]. Causal knowledge may yet be important though, as extreme political positions have been shown to be relaxed when people holding such views are prompted to provide causal explanations to support their views[44]. Fostering higher levels of knowledge about climate change issues may serve to reduce the extent of polarization[45]. Ultimately, the tension between knowledge, values, and polarization remains an interesting issue that warrants further research.

### Relationship to climate change attitudes

An important question left open by our research is how a causal knowledge intervention, which we have shown to influence people’s economic decision-making behavior, relates to more general attitudes about climate change mitigation. It is now established that knowledge about the causes of climate change is a key driver of climate change acceptance among the general public[46–47]. At a more practical level, relevant causal knowledge about climate change has also been shown to increase people’s intention to engage in mitigation behavior[48], as well as levels of support for implementing climate change mitigation policies[49–50]. It follows that possessing relevant causal knowledge about climate change dynamics—and the pivotal role that human (economic) activities play in driving these dynamics—has the capacity to influence how people think about the climate system, and importantly, consider how their actions might adversely affect it[1]. At minimum, it seems that equipping people with relevant causal knowledge will make achieving collective action on climate change more likely to happen.

### Conclusions

The results of our study show that when people are made aware of the economic ramifications of climate change, they alter the way they make decisions about satisfying an economic goal. By showing a beneficial effect of causal knowledge on people’s decision-making, our study reinforces the utility of knowledge-based interventions in advancing public debate on climate change mitigation. Highlighting the critical role of human CO_2_ emissions in causing climate change coupled with the economic risks posed by climate change produced a dramatic reduction in the economic targets people set. Specifically, people appeared able to forego short-term economic gains if they knew that doing so would increase the likelihood that continued growth over the long-term could be achieved. This resulted in long-term economic sustainability and significantly better environmental outcomes (viz. less accumulated CO_2_ and less warming) compared to when no causal information was provided. Given the role of causal knowledge in evaluating the effectiveness of mitigation strategies and their socio-economic implications[43,50], we believe our results reinforce the idea that relevant knowledge improves people’s decision-making with regards to climate change mitigation.

## Supporting information

S1 Text Participant instructions and additional analyses. (DOCX)

S1 File Data from Experiment 1. (SAV)

S2 File Data from Experiment 2. (SAV)

## References

[1] Newell BR, McDonald RI, Brewer M, Hayes BK. The psychology of environmental decisions. Annu Rev Environ Resour. 2014;39(1):443–467.

[2] Sterman JD. All models are wrong: reflections on becoming a systems scientist. Syst Dyn Rev. 2002;18(4):501–531.

[3] Osman M. Controlling uncertainty: a review of human behavior in complex dynamic environments. Psychol Bull. 2010;136(1):65–86. doi: 10.1037/a0017815 20063926 10.1037/a0017815

[4] Sloman SA, Fernbach PM. Human representation and reasoning about complex causal systems. Inf Knowl Syst Manage. 2011;10(1–4): 85–99.

[5] Keil FC. Folkscience: coarse interpretations of a complex reality. Trends Cogn Sci. 2003;7(8):368–373. 12907233 10.1016/s1364-6613(03)00158-x

[6] Rozenblit L, Keil FC. The misunderstood limits of folk science: an illusion of explanatory depth. Cogn Sci. 2002;26(5):521–562. doi: 10.1207/s15516709cog2605_1 21442007 10.1207/s15516709cog2605_1 PMC3062901

[7] Cronin MA, Gonzalez C, Sterman JD. Why don’t well-educated adults understand accumulation? a challenge to researchers, educators, and citizens. Organ Behav Hum Decis Process. 2009;108(1):116–130.

[8] Pidgeon N, Fischhoff B. The role of social and decision sciences in communicating uncertain climate risks. Nat Clim Chang. 2011;1(1):35–41.

[9] Weber EU, Stern PC. Public understanding of climate change in the United States. Am Psychol. 2011;66(4):315–328. doi: 10.1037/a0023253 21553956 10.1037/a0023253

[10] Bostrom A, Morgan MG, Fischhoff B, Read D. What do people know about global climate change? 1. mental models. Risk Anal. 1994;14(6):959–970.

[11] Kempton W. Lay perspectives on global climate change. Glob Environ Change. 1991;1(3):183–208.

[12] Read D, Bostrom A, Morgan MG, Fischhoff B, Smuts T. What do people know about global climate change? 2. survey studies of educated laypeople. Risk Anal. 1994;14(6):971–982. 10.1111/j.1539-6924.2010.01448.x PMC6170370 20649942

[13] Reynolds TW, Bostrom A, Read D, Morgan MG. Now what do people know about global climate change? survey studies of educated laypeople. Risk Anal. 2010;30(10):1520–1538. doi: 10.1111/j.1539-6924.2010.01448.x 20649942 10.1111/j.1539-6924.2010.01448.x PMC6170370

[14] Sterman JD, Booth Sweeney L. Understanding public complacency about climate change: adults’ mental models of climate change violate conservation of matter. Clim Change. 2007;80(4):213–238.

[15] Sterman JD. Risk communication on climate: mental models and mass balance. Science. 2008;322(5901):532–533. doi: 10.1126/science.1162574 18948524 10.1126/science.1162574

[16] Dutt V, Gonzalez C. Human control of climate change. Clim Change. 2012;111(3):497–518.

[17] Guy S, Kashima Y, Walker I, O’Neill S. Comparing the atmosphere to a bathtub: effectiveness of analogy for reasoning about accumulation. Clim Change. 2013;121(4):579–594.

[18] Moxnes E, Saysel AK. Misperceptions of global climate change: information policies. Clim Change. 2009;93(1–2):15–37.

[19] Newell BR, Kary A, Moore C, Gonzalez C. Managing the budget: stock-flow reasoning and the CO_2_ accumulation problem. Top Cogn Sci. 2016;8(1):138–159. doi: 10.1111/tops.12176 26695816 10.1111/tops.12176

[20] Booth Sweeney L, Sterman JD. Bathtub dynamics: initial results of a systems thinking inventory. Syst Dyn Rev. 2000;16(4):249–286.

[21] Dutt V, Gonzalez C. Decisions from experience reduce misconceptions about climate change. J Environ Psychol. 2012;32(1):19–29.

[22] Diehl E, Sterman JD. Effects of feedback complexity on dynamic decision making. Organ Behav Hum Decis Process. 1995;62(2):198–215.

[23] Sterman JD. Modeling managerial behavior: misperceptions of feedback in a dynamic decision environment. Manage Sci. 1989;35(3):321–339.

[24] O'Connor RE, Bord RJ, Yarnal B, Wiefek N. Who wants to reduce greenhouse gas emissions? Soc Sci Q. 2002;83(1):1–17.

[25] Brulle RJ, Carmichael J, Jenkins JC. Shifting public opinion on climate change: An empirical assessment of factors influencing concern over climate change in the U.S., 2002–2010. Clim Change. 2012;114(2), 169–188.

[26] Kahn ME, Kotchen MJ. Business cycle effects on concern about climate change: The chilling effect of recession. Climate Change Economics. 2011;2(3),257–273.

[27] Rogelj J, McCollum DL, Reisinger A, Meinshausen M, Riahi K. Probabilistic cost estimates for climate change mitigation. Nature. 2013;493(7430):79–83. doi: 10.1038/nature11787 23282364 10.1038/nature11787

[28] Meinshausen M, Raper SCB, Wigley TML. Emulating coupled atmosphere-ocean and carbon cycle models with a simpler model, MAGICC6 –Part 1: Model description and calibration. Atmos Chem Phys. 2011;11(4):1417–1456.

[29] Bodman RW, Rayner PJ, Karoly DJ. Uncertainty in temperature projections reduced using carbon cycle and climate observations. Nat Clim Chang. 2013;3(8):725–729.

[30] Lagnado DA, Sloman SA. The advantage of timely intervention. J Exp Psychol Learn Mem Cogn. 2004;30(4):856–876. doi: 10.1037/0278-7393.30.4.856 15238029 10.1037/0278-7393.30.4.856

[31] Rottman BM, Keil FC. Causal structure learning over time: observations and interventions. Cogn Psychol. 2012;64(1–2):93–125. doi: 10.1016/j.cogpsych.2011.10.003 22155679 10.1016/j.cogpsych.2011.10.003 PMC3309528

[32] Steyvers M, Tenenbaum JB, Wagenmakers EJ, Blum B. Inferring causal networks from observations and interventions. Cogn Sci. 2003;27(3):453–489.

[33] Gonzalez C, Lerch JF, Lebiere C. Instance-based learning in dynamic decision making. Cogn Sci. 2003;27(4):591–635.

[34] Burns BD, Vollmeyer R. Goal specificity effects on hypothesis testing in problem solving. Q J Exp Psychol. 2002;55(1):241–261. 10.1080/02724980143000262 11873850

[35] Hagmayer Y, Meder B, Osman M, Mangold S, Lagnado D. Spontaneous causal learning while controlling a dynamic system. Open Psychol J. 2010;3(1):145–162.

[36] Hagmayer Y, Meder B. Repeated causal decision making. J Exp Psychol Learn Mem Cogn. 2013;39(1):33–50. doi: 10.1037/a0028643 22686840 10.1037/a0028643

[37] Waldmann MR. Predictive versus diagnostic causal learning: evidence from an overshadowing paradigm. Psychon Bull Rev. 2001;8(3):600–608. 11700912 10.3758/bf03196196

[38] Crump MJC, McDonnell JV, Gureckis TM. Evaluating Amazon's Mechanical Turk as a tool for experimental behavioral research. PLoS ONE. 2013;8(3):e57410 doi: 10.1371/journal.pone.0057410 23516406 10.1371/journal.pone.0057410 PMC3596391

[39] Buhrmester M, Kwang T, Gosling SD. Amazon’s Mechanical Turk: A new source of inexpensive, yet high-quality, data? Perspect Psychol Sci. 2011;6(1):3–5. doi: 10.1177/1745691610393980 26162106 10.1177/1745691610393980

[40] Rottman BM, Hastie R. Reasoning about causal relationships: Inferences on causal networks. Psychol Bull. 2014;140(1):109–139. doi: 10.1037/a0031903 23544658 10.1037/a0031903 PMC3988659

[41] Dörner D. The logic of failure. Philos Trans R Soc Lond B Biol Sci. 1990;327(1):463–473. 1970892 10.1098/rstb.1990.0089

[42] Moxnes E. Not only the tragedy of the commons: misperceptions of feedback and policies for sustainable development. Syst Dyn Rev. 2000;16(4):325–348.

[43] Bain PG, Hornsey MJ, Bongiorno R, Jeffries C. Promoting pro-environmental action in climate change deniers. Nat Clim Chang. 2012;2(8):603–603.

[44] Fernbach PM, Rogers T, Fox CR, Sloman SA. Political extremism is supported by an illusion of understanding. Psychol Sci. 2013;24(6):939–946. doi: 10.1177/0956797612464058 23620547 10.1177/0956797612464058

[45] Guy S, Kashima Y, Walker I, O'Neill S. Investigating the effects of knowledge and ideology on climate change beliefs. Eur J Soc Psychol. 2014;44(5):421–429.

[46] Clark D, Ranney MA, Felipe J. Knowledge helps: Mechanistic information and numeric evidence as cognitive levers to overcome stasis and build public consensus on climate change. In: Knauff M, Pauen M, Sebanz N, Wachsmuth I, editors. Proceedings of the 35th annual meeting of the cognitive science society. Austin: Cognitive Science Society; 2013. pp. 2070–2075.

[47] Ranney MA, Clark D, Reinholz DL, Cohen S. Changing global warming beliefs with scientific information: knowledge, attitudes, and RTMD (reinforced theistic manifest destiny theory). In: Miyake A, Peebles D, Cooper RP, editors. Proceedings of the 34th annual meeting of the cognitive science society. Austin: Cognitive Science Society; 2012. pp. 2228–2233.

[48] O'Connor RE, Bord RJ, Fisher A. Risk perceptions, general environmental beliefs, and willingness to address climate change. Risk Anal. 1999;19(3):461–471.

[49] Bord RJ, O'Connor RE, Fisher A. In what sense does the public need to understand global climate change? Public Underst Sci. 2000;9:205–218.

[50] Bostrom A, O’Connor RE, Böhm G, Hanss D, Bodi O, Ekström F, et al Causal thinking and support for climate change policies: international survey findings. Glob Environ Change. 2012;22(1):210–222.

