# Correction: Causal knowledge promotes behavioral self-regulation: An example using climate change dynamics

**DOI:** 10.1371/journal.pone.0228445

**Published:** 2020-01-24

**Authors:** David K Sewell, Peter J Rayner, Daniel B Shank, Sophie Guy, Simon D. Lilburn, Saam Saber, Yoshihisa Kashima

In the Task overview: Managing a dynamic human-climate system subsection of the Introduction, there is an error in equation 4. There is a factor of τ that is missing from the denominator of the first term that appears on the right-hand side of the equation. Please view the complete, correct equation here:

$$\Delta T_{i}=\frac{\sigma c_{i}}{\tau c_{pre}}-\frac{T_{i}}{\tau }$$
