# Supplementary material for: Causal knowledge promotes behavioral self-regulation: An example using climate change dynamics
Source: PLoS One. 2017 Sep 7;12(9):e0184480. doi: 10.1371/journal.pone.0184480 (PMC5589253; doi:10.1371/journal.pone.0184480)
Supplement: S1 Text — (DOCX) [file pone.0184480.s001.docx]

**Supporting Information for Sewell, Rayner, Shank, Guy, Lilburn, Saber, and Kashima (2017). Causal knowledge promotes behavioral self-regulation: An example using climate change dynamics.**

**Participant Instructions**

Participants in the Informed condition of Experiment 1 received the following written instructions, reproduced here in full, which they were free to refer to throughout the experiment. The figure in the instructions shows the response screen presented to participants on each trial. Participants in the Uninformed condition of Experiment received the same instructions, but without the information in the black-bordered box.

“*This task explores the relationship between economic decisions in a dynamic environment that is sensitive to climate.*

*You are playing the role of a policy director in charge of setting economic targets for the year ahead. On each trial, you will be required to set a productivity target by clicking somewhere in the bar at the top of the screen. You will also be given information about the Current Economic Index, and two key features of the climate. These are the concentration of CO2 in the atmosphere, and the average global temperature in degrees Celsius.*

*
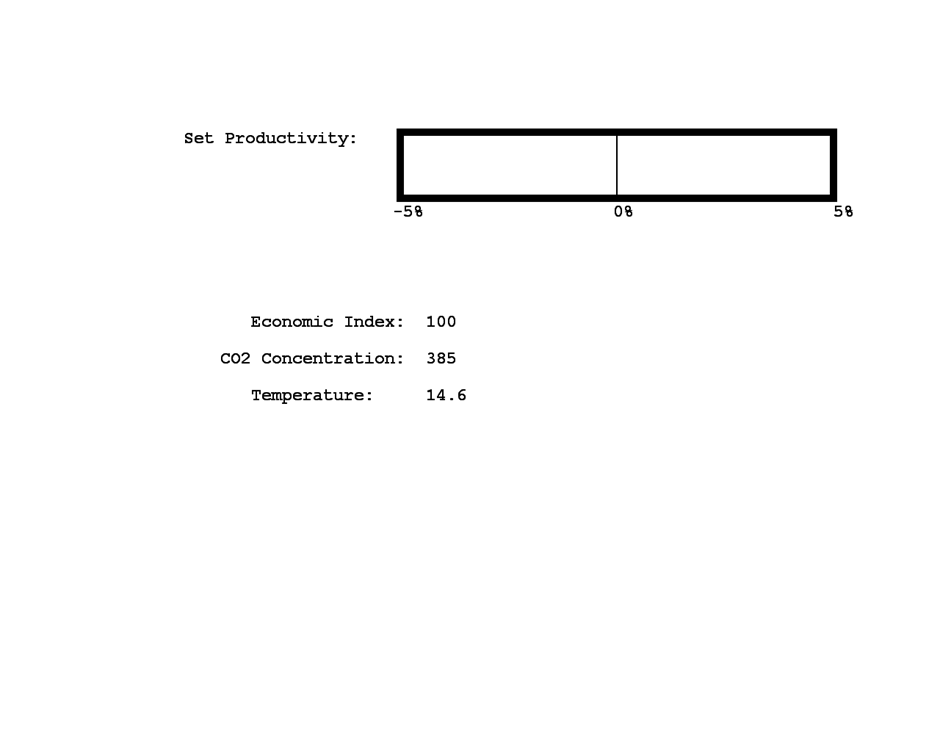
*

*The productivity target can range from -5% to +5% of the current Economic Index.*

*After confirming your productivity target, you will receive feedback. You will be told about changes in the Economic Index, the CO2 concentration, and the Temperature.*

*You will also be given feedback on the Economic Growth Rate (the change in the Economic Index, expressed as a percentage of the previous year).*

*Economic productivity affects CO2 concentration, which in turn affects Temperature. Temperature increases make it increasingly difficult to achieve economic growth. Due to time lags in the climate system, the effects of CO2 on economic growth will only be felt after a considerable delay, after which they will be difficult to reverse. Hence, it is advisable to keep CO2 concentration from escalating too high.*

***Your aim is to maximize the overall Economic Index.***

***Aim to at least double the Economic Index by the end of the experiment.***

*The experiment comprises 250 trials. Take short breaks when necessary.*

*If you have any questions, please ask the experimenter now.*

*Thank you for your participation!”*

Participant instructions also differed depending on the context in which the task was presented. For participants in the climate change context, the variables were labelled ‘economic index’, ‘CO_2_ concentration’, and ‘temperature’, as shown above, and discussed in the main article. For participants in the other condition, the task was presented in the context of managing a fictitious population of bacteria, where the variable labels were changed to reflect the different cover story (i.e., ‘bacterial population’, ‘sample dispersion’, and ‘immunological resistance’ replaced ‘economic index’, ‘CO_2_ concentration’, and ‘temperature’), and the explicit goal of the task was relabelled appropriately (i.e., participants were told to aim to double the size of the bacterial population by the end of the experiment). The instructions were otherwise identical in all other respects. To assess the effect of cover story, independent of system dynamics, the same underlying causal model was used regardless of cover story.

**Additional Statistical Analyses**

Owing to the large number of dependent variables analyzed in the study, only a subset of the most relevant results were presented in the main article. Here, we report additional analyses on the participant response data to supplement those reported in the main text.

For participants in Experiment 1, the primary factor influencing their responding—the economic targets they set—was Information Condition. Informed participants set more conservative economic targets than Uninformed participants. In addition to the main effect reported in the main text—our principal finding—there was also an interaction between Cover Story and Information Condition, *F* (1, 96) = 4.72, *MS_e_*= .001, *p* = .03, *η_p_^2^* = .05, reflecting the higher economic targets set by Uninformed participants, but not Informed participants, in the Bacteria cover story condition. However, we believe this interaction may be a spurious effect, as it did not replicate in Experiment 2 with our online sample. In Experiment 2, there was no effect of Cover Story on participant responding, *F* (1, 108) = 0.99, *p* = .32, nor an interaction between Cover Story and Information Condition, *F* (1, 108) = 1.90, *p* = .17.

As noted in the main text, for both experiments, there were significant effects of Information Condition on responding in both experiments. In turn, this resulted in main effects of Information Condition on both CO_2_ and temperature for both experiments. As is evident in Fig 3 and 4 of the main text, these variables display different trajectories as a function of Information Condition. In Experiment 1, there are significant interactions between Information Condition and Trial Epoch for CO_2_, *F* (9, 864) = 33.97, *MS_e_*=5132.76, *p* < .001, *η_p_^2^* = .26, and temperature, *F* (9, 864) = 51.28, *MS_e_*= 0.57, *p* < .001, *η_p_^2^* = .35. For Experiment 1, there were also significant three-way interactions between Information Condition, Trial Epoch, and Cover Story—arising from the (likely) spurious Cover Story by Information Condition interaction effect on responding—for both CO_2_, *F* (9, 864) = 3.85, *MS_e_*= 5132.76, *p* < .001, *η_p_^2^* = .04, and temperature, *F* (9, 864) = 4.98, *MS_e_*= .57, *p* < .001, *η_p_^2^* = .05. For Experiment 2, there was no such three-way interaction for either CO_2_, *F* (9, 972) = 0.90, *p* = .53, or temperature, *F* (9, 972) = 1.16, *p* = .32.

**Additional Details of Optimal Response Profile Analysis**

Given the explicit economic goals of the task, it is of interest to determine whether there is an optimal way of managing the human-climate system. That is, identifying a response profile that most successfully achieves the task goal of maximizing the overall economic index whilst also attempting to (at least) double the economic index after 250 trials. In our task though, identifying an optimal response profile is difficult because the behavior of the human-climate system, discussed in the main text, involves non-linearities, time delays, and complex interactions among system variables. As such, achieving an analytic solution to this question is not possible. In light of this, we adopted a constrained optimization approach where we divided the task into 25 equally-sized response epochs. A standard simplex algorithm was used to adjust economic growth targets in each response epoch until the algorithm converged on the response profile that maximized the average of (a) the mean economic index over the course of the task, and (b) the value of the economic index at a time point slightly beyond the life of the task (i.e., at trial 300)—see main text for discussion.

Although our optimization approach allows us to address questions about the best way of performing the task, it is not without caveats. For one, the optimization process is sensitive to information that is effectively hidden from participants in the study. For example, the optimization algorithm, unlike participants in our study, does not need to learn the effect different economic growth targets have on the system variables. On the contrary, the optimization routine has full knowledge of these effects in addition to their interaction with time delays within the system. Our initial exploratory analyses revealed highly implausible response profiles that exploited the hidden structure of the human-climate system, the bulk of which involved aggressive reduction of the economic index in the middle part of the task followed by very rapid ramping up of economic growth targets toward the end of the task. The rationale behind dramatically reducing the economic index during the early and middle parts of the task—sometimes to zero—was to minimize the penalty warming has on economic growth toward the end of the task (see Equation 2 in the main text). Because of time delays in the system, warming that occurs in the latter part of the task is effectively unpenalized, resulting in inflated economic index values. (This aspect of the system produces an “end-of-task” ramping artifact in the optimization analysis. By evaluating the economic index at trial 300, rather than trial 250, we were able to reduce its impact, but were unable to eliminate it completely.)

To avoid implausible response profiles that aggressively shrunk the economic index partway through the task, we constrained economic growth rates to be such that the cumulative change in the economic index had to at least keep pace with linear growth toward an economic index of 200 at trial 250. Because we also found that estimates for the early response epochs were highly volatile, resulting in convergence problems, we constrained the form of the response function to be exponential (see Equation 5 in the main text). This proved to minimize the influence of artifacts associated with the optimization analysis, resulting in highly stable optimization, whilst also producing response profiles that were not qualitatively dissimilar from the kinds of response profiles we observed empirically.
